# Reply to: Letter to the Editor — Extending the Phase-dependent VEGF Framework to Post-Intracerebral Hemorrhage Epileptogenesis

**DOI:** 10.31083/RN57522

**Published:** 2026-09-23

**Authors:** Yunlian Niu, Xiaoqian Wang, Yichen Huang, Xuemei Zhang, Yijie You, Peiyuan Ding, Fengbing Sun, Jian Zhang

**Affiliations:** ^1^Department of Neurology, Chongming Hospital Affiliated to Shanghai University of Medicine and Health Sciences, 202150 Shanghai, China; ^2^Department of Neurosurgery, Chongming Hospital Affiliated to Shanghai University of Medicine and Health Sciences, 202150 Shanghai, China; ^3^Ward 4 of the Stroke Center, Chongming Hospital Affiliated to Shanghai University of Medicine and Health Sciences, 202150 Shanghai, China

To the Editor,

We thank the authors for their thoughtful, carefully argued letter regarding our review on the dual role of vascular endothelial growth factor (VEGF) in intracerebral hemorrhage (ICH) [1]. We fully share their view that post-ICH seizures and epileptogenesis constitute a clinically important yet often overlooked dimension of long-term outcomes after ICH, and that the phase-dependent VEGF framework we described carries underappreciated implications for this endpoint. Their perspective adds substantial translational value to the topic, and we are pleased to respond to the key points they raise.

First, the authors rightly draw a direct line between blood-brain barrier (BBB) disruption—a central mechanism in our model—and established mediators of post-injury epileptogenesis. Our review was deliberately focused on neurovascular barrier integrity, vasogenic edema, glymphatic function and angiogenic repair, and did not extend its scope to cortical network excitability and seizure risk. As the authors detail, vascular endothelial growth factor receptor (VEGFR)-2/Src-mediated vascular endothelial (VE)-cadherin phosphorylation, internalization of tight junction proteins such as claudin-5 and occludin, and matrix metalloproteinase (MMP)-driven basement membrane degradation do more than drive edema formation [2]. These events also allow albumin extravasation into the neuropil, which triggers astrocytic transforming growth factor beta (TGF-β) receptor signaling, impairs extracellular potassium buffering via Kir4.1 downregulation, and ultimately promotes epileptiform activity [3,4]. This mechanistic convergence is biologically compelling, and we agree it represents a meaningful, underexplored extension of the phase-dependent model we proposed.

Second, we also greatly appreciate the authors’ note of caution about the temporal overlap between the proposed subacute-to-chronic reparative window (days 3–14) and the latent period of epileptogenesis, as well as their nuanced discussion of VEGFR-3 selectivity. In our review, we positioned the VEGF-C/VEGFR-3 axis as a preferentially reparative pathway, on the basis that it signals predominantly through phosphoinositide 3-kinase/protein kinase B (PI3K/Akt) and couples only weakly to the pro-permeability Src cascade. The authors are correct that this selectivity is relative, not absolute. Proteolytically processed, mature VEGF-C can activate VEGFR-2 in addition to VEGFR-3, and VEGFR-2/VEGFR-3 heterodimers have been identified on angiogenic sprouts [5,6].

Whether these receptor interactions meaningfully alter cortical excitability in the post-ICH brain remains an open question. As the authors acknowledge, much of the existing evidence linking VEGF-C/VEGFR-3 upregulation to epileptogenesis comes from status epilepticus and mesial temporal lobe epilepsy models [7,8], which differ substantially from ICH in injury pattern, anatomical distribution and inflammatory context. Even so, their observation that this signaling axis is dynamically regulated during the very window in which therapeutic activation is proposed is an important safety consideration that deserves formal investigation in future ICH studies.

Third, we wholeheartedly agree that conventional trial endpoints—hematoma volume, perihematomal edema, and 90-day functional scales—are insensitive to delayed seizure outcomes. Late seizures after ICH accumulate gradually over years, with cumulative risk reaching nearly 12% at 5 years [9]. A therapy that improves short-term functional outcomes but modifies long-term seizure risk could therefore be misclassified as successful under standard trial designs.

The practical methodological suggestions the authors put forward are well grounded and highly actionable: distinguishing acute symptomatic seizures, late unprovoked seizures and post-ICH epilepsy as separate outcomes; adopting a minimum 24‑month follow‑up window; in this cohort [6], 10.0% cumulative risk of late seizures was observed at 2 years, representing roughly 85% of the total 11.8% 5‑year cumulative late‑seizure events; incorporating continuous electroencephalography (EEG) monitoring when clinically indicated; and stratifying risk using the cortical involvement, age <65 years, hematoma volume >10 mL, and early seizure (CAVE) score with explicit accounting for cortical involvement [9,10,11]. Adding these elements to future preclinical and translational studies of VEGF-directed therapies would markedly strengthen their clinical relevance.

All in all, the authors make a very strong case for extending the phase-dependent VEGF framework to include electrical network remodeling and seizure-related outcomes. Our review centered on neurovascular and glymphatic mechanisms of injury and repair, and we recognize that post-ICH epileptogenesis is a clinically relevant downstream consequence that intersects with multiple pathways we described. Integrating seizure endpoints, longer follow-up and risk stratification into future work will help clarify whether VEGF-modulating therapies optimized for neurovascular repair also carry effects on long-term cortical excitability.

We thank the authors again for their careful, constructive contribution. It deepens the interpretation of our work and adds an important translational perspective to the field.
